# PD99 Effectiveness And Safety Of Bioelectrical Stimulation Techniques In Fibromyalgia: A Systematic Review

**DOI:** 10.1017/S0266462324003453

**Published:** 2025-01-07

**Authors:** Yolanda Álvarez-Pérez, Andrea Duarte-Díaz, Amado Rivero-Santana, Vanesa Ramos García, Violeta Cazaña-Pérez, Inés Llamas-Ramos, Elisa Trujillo, Teresa Téllez-Santana, Ángela Brocalero, Lilisbeth Perestelo-Pérez

## Abstract

**Introduction:**

Fibromyalgia, a musculoskeletal ailment of unknown origin, profoundly affects quality of life. Emerging bioelectrical stimulation techniques, including transcranial magnetic stimulation (TMS), transcranial direct current stimulation (tDCS), and pulsed low-frequency magnetic field stimulation (PEMF), show promise in short-term pain alleviation. This study aimed to rigorously evaluate the effectiveness and safety of these techniques in treating fibromyalgia.

**Methods:**

A systematic review (SR) of available literature on the effectiveness and safety of bioelectrical stimulation techniques was carried out according to the Cochrane Collaboration methodology and PRISMA reporting guideline. Evaluated studies included SRs (with or without meta-analyses) and randomized controlled trials (RCTs) published after the SRs. SRs were appraised with the AMSTAR-2 tool and RCTs were assessed with version two of the Cochrane Collaboration risk-of-bias tool for randomized trials. The findings were synthesized narratively. In the absence of SRs with meta-analyses for specific techniques, we conducted a meta-analysis for each available outcome measure, including pain, fatigue, symptom severity, quality of life, anxiety, and depression.

**Results:**

Seven SRs incorporating 35 RCTs were included. Two SRs evaluated TMS effectiveness, while five focused on tDCS. Additionally, 17 RCTs were included: two on repetitive TMS, six on tDCS, and eight on PEMF (three assessing targeted PEMF). General confidence in the SR results varied, with most having critically low confidence. Three additional RCTs were rated as low risk of bias, seven were rated as unclear risk of bias, and the remaining seven were rated as high risk of bias. A meta-analysis covered additional RCTs on PEMF and assessed pain intensity, symptom severity, general health-related quality of life, and fibromyalgia-related quality of life.

**Conclusions:**

Overall, the results suggest that repetitive TMS, tDCS, and PEMF could improve pain and quality of life in patients with fibromyalgia. It is, however, necessary to conduct high quality studies to demonstrate the clinical relevance of these effects. While the techniques evaluated appear to be safe, mild adverse effects involving the area of stimulation may occur.

